# TGR5 Activation Ameliorates Mitochondrial Homeostasis *via* Regulating the PKCδ/Drp1-HK2 Signaling in Diabetic Retinopathy

**DOI:** 10.3389/fcell.2021.759421

**Published:** 2022-01-14

**Authors:** Meng-Yuan Zhang, Lingpeng Zhu, Xinhua Zheng, Tian-Hua Xie, Wenjuan Wang, Jian Zou, Yan Li, Hong-Ying Li, Jiping Cai, Shun Gu, Yong Yao, Ting-Ting Wei

**Affiliations:** ^1^ Department of Ophthalmology, The Affiliated Wuxi People’s Hospital of Nanjing Medical University, Wuxi, China; ^2^ Center of Clinical Research, The Affiliated Wuxi People’s Hospital of Nanjing Medical University, Wuxi, China; ^3^ Department of Ophthalmology, The Affiliated Wuxi No. 2 People’s Hospital of Nanjing Medical University, Wuxi, China

**Keywords:** diabetic retinopathy, TGR5, mitochondrial fission, mitophagy, mitochondrial homeostasis

## Abstract

**Background:** Diabetic retinopathy (DR) is one of the most important microvascular diseases of diabetes. Our previous research demonstrated that bile acid G-protein-coupled membrane receptor (TGR5), a novel cell membrane receptor of bile acid, ameliorates the vascular endothelial cell dysfunction in DR. However, the precise mechanism leading to this alteration remains unknown. Thus, the mechanism of TGR5 in the progress of DR should be urgently explored.

**Methods:** In this study, we established high glucose (HG)-induced human retinal vascular endothelial cells (RMECs) and streptozotocin-induced DR rat *in vitro* and *in vivo*. The expression of TGR5 was interfered through the specific agonist or siRNA to study the effect of TGR5 on the function of endothelial cell *in vitro*. Western blot, immunofluorescence and fluorescent probes were used to explore how TGR5 regulated mitochondrial homeostasis and related molecular mechanism. The adeno-associated virus serotype 8-shTGR5 (AAV8-shTGR5) was performed to evaluate retinal dysfunction *in vivo* and further confirm the role of TGR5 in DR by HE staining, TUNEL staining, PAS staining and Evans Blue dye.

**Results:** We found that TGR5 activation alleviated HG-induced endothelial cell apoptosis by improving mitochondrial homeostasis. Additionally, TGR5 signaling reduced mitochondrial fission by suppressing the Ca^2+^-PKCδ/Drp1 signaling and enhanced mitophagy through the upregulation of the PINK1/Parkin signaling pathway. Furthermore, our result indicated that Drp1 inhibited mitophagy by facilitating the hexokinase (HK) 2 separation from the mitochondria and HK2-PINK1/Parkin signaling. *In vivo*, intraretinal microvascular abnormalities, including retinal vascular leakage, acellular capillaries and apoptosis, were poor in AAV8-shTGR5-treated group under DR, but this effect was reversed by pretreatment with the mitochondrial fission inhibitor Mdivi-1 or autophagy agonist Rapamycin.

**Conclusion:** Overall, our findings indicated that TGR5 inhibited mitochondrial fission and enhanced mitophagy in RMECs by regulating the PKCδ/Drp1-HK2 signaling pathway. These results revealed the molecular mechanisms underlying the protective effects of TGR5 and suggested that activation of TGR5 might be a potential therapeutic strategy for DR.

## Introduction

Diabetic retinopathy (DR) is one of the most frequent microvascular complications of diabetes mellitus (DM) and the leading cause of blindness in the working-age population (Daskivich et al., 2017). The number of patients with DM is currently estimated to be 463 million. By the year 2040, the number of diabetics worldwide is expected to rise to 642 million, and about one-third of patients may suffer a diabetic retinal disease (Weber et al., 2016). Considering the current lack of medical therapy for DR, insights into underlying mechanisms are important. Many types of retinal cells, such as endothelial cells, pericytes, ganglion cells and Müller cells, die in advanced complications of DM (Volpe et al., 2018). Amongst them, retinal endothelial cells (RMECs), crucial regulators of retinal capillary beds, obtain nutrients and excrete wastes (Antonetti et al., 2021). Basic and clinical research indicated that the endothelium is the first to be affected during DR (Economopoulou et al., 2005). Hyperglycaemia causes endothelial apoptosis and pericyte loss, thereby promoting the formation of acellular capillaries and leading to blood–retina barrier (BRB) breakdown (Roy and Kim, 2021). Moreover, the anti-vascular endothelial growth factor agents such as aflibercept and ranibizumab represent antibody is one of the most effective tools for DR treatment, suggesting its key role for the regulation of endothelial function upon DR (Giurdanella et al., 2015; Varadarajan et al., 2020). Therefore, we can speculate that the improvement in the retinal endothelial function may retard the progression of DR.

In peripheral blood vessels, vascular endothelial cells directly come into contact and interact with high glucose (HG). Persistent hyperglycaemia induces metabolic dysfunction in endothelial cells (Yamamoto and Yamamoto, 2013). Mitochondrial function is strongly linked to cell division, growth and death. As a major site of energy production, mitochondria have a crucial role in energy conversion and metabolism. The mitochondrial quality control (MQC) is closely related to the cellular microenvironment and functions (Bock and Tait, 2020). Mitochondria are double-membrane organelles with high fluidity and can synergistically undergo fission and fusion under the precise regulation of a variety of proteins. Three proteins, mitofusin1 (Mfn1), mitofusin2 (Mfn2) and optic atrophy 1 (OPA1), are involved in mitochondrial fusion. Mitochondrial fission is mediated by dynamin-related protein 1 (Drp1) and its receptors, including mitochondrial fission factor (MFF) and fission 1 (Fis1) (Ferrington et al., 2020). The formation and maintenance of the mitochondrial network have an essential role in maintaining mitochondrial functions. Mitochondrial fusion and fission normally exist in a dynamic equilibrium. An imbalance in mitochondrial dynamics can cause various diseases in humans (Sabouny and Shutt, 2020). Patients with DM have significantly increased ratio of fragmented mitochondria compared with the healthy population (Ma et al., 2012). The knockdown of Fis1 or Drp1 attenuates HG-induced ROS accumulation and endothelial cell damage (Kim et al., 2020). After myocardial ischemia/reperfusion (I/R) injury, mitochondrial disorders in the endothelium initiate coronary microvascular dysfunction (Marin-Garcia and Akhmedov, 2016). Conversely, fusion allows normal and dysfunctional mitochondria to redistribute proteins and anchor them together through a “fusion device”, thereby maintaining the integrity of mtDNA and restoring cell viability (Wang et al., 2020). These results suggest that the excess production of damaged mitochondria may be an important cause of endothelial injury.

Under physiological states, mitophagy eliminates damaged mitochondria through the induction of lysosome-dependent degradation (Yu et al., 2016). Mitophagy provides mitochondrial repair, thus promoting mitochondrial quality control (MQC) MQC to meet the cell’s energy and material requirements. Mitochondrial homeostasis relies on mitochondrial biogenesis and dynamics (fission and fusion) and degradation through mitophagy (Yamashita and Kanki, 2017; Dehdashtian et al., 2018). The overexpression of autophagy-associated proteins, such as ATG5, ATG12, and LC3B, has been shown to remarkably prolong the life of endothelial cells (Lin et al., 2019). Impaired autophagy leads to endothelial dysfunction and accelerates the progression of vascular calcification and atherosclerosis (Spengler et al., 2020). Mitophagy suppresses ROS production by orchestrating cellular redox status, whereas defects of autophagy are closely associated with severe oxidative stress and abnormal vascular functions (Pradeepkiran and Reddy, 2020). Therefore, we believe that enhancing mitophagy and improving endothelial mitochondrial function may be a useful strategy for diseases associated with vascular abnormalities.

The mitochondrial quality is crucially linked to energy metabolism. Recently, bile acids have emerged as pivotal signaling molecules for the regulation of glucose metabolism, lipid homeostasis, as well as energy metabolism, which exerts multiple biological effects by binding to its receptors, the bile acid nuclear receptor Farnesoid X receptor (FXR) and the membrane G-protein-coupled bile acid receptor (TGR5). Moreover, dysregulation of signal molecules in bile acid can result in lipid, glucose dysmetabolism and the gut dysbiosis. TGR5 has been reported to play a critical role in energy metabolism, while the direct relationship of bile acids and microvascular abnormalities in DR is rarely reported The bile acid G-protein-coupled membrane receptor (TGR5), a novel cell membrane receptor of bile acid, has been reported to play a critical role in energy metabolism (Pols et al., 2011; van Nierop et al., 2017). Mitochondria are the most important organelles in the regulation of energy generation. That is to say, mitochondrial quality is crucially linked to energy metabolism. In our previous studies, we found that the TGR5 agonist significantly alleviates DR-induced retinal vascular leakage, but the precise mechanisms of action are still unknown (Zhu et al., 2020). Hence, this study is designed to examine the effect of TGR5 on mitochondrial homeostasis in RMECs. Our results show that TGR5 inhibits mitochondrial fission and/or enhances mitophagy by regulating Drp1-HK2 signaling, thereby lowering endothelial dysfunction and alleviating DR progression.

## Materials and Methods

### Materials and Reagents

Mdivi-1, 3-bromopyruvic acid, INT-777, 3-methyladenine, Rapamycin, BAPTA-AM, and Z-VAD (OMe)-FMK were procured from MedChemExpress (NJ, United States). MitoBright LT Red was obtained from DOJINDO (Kumamoto, Japan). Glucose, streptozocin (STZ) and Evans Blue were obtained from Sigma-Aldrich (St. Louis, United States). Fluo-4 AM, Cell counting kit-8 (CCK8), JC1 assay kit and cell mitochondria isolation kit were obtained from Beyotime (Shanghai, China). The TUNEL assay and haematoxylin-eosin (HE) staining kits were obtained from Roche (Mannheim, Germany) and BOSTER (Wuhan, China), respectively. The primary antibodies used were as follows: PKC δ, Phospho-PKCδ, HK2, LC3B, P62, DRP1 (Abcam, Cambridge, United States); Parkin, PINK1, Phospho-DRP1, Cleaved caspase-3 (Cell Signaling Technology, Danvers United States), Bax, β-actin, COX4 (Proteintech, Wuhan, China); and TOMM20 (Santa, CA, United States). information on reagents and antibodies is shown in Supplementary Tables S1, S2.

### Cell Culture

Human retinal microvascular endothelial cells (HRMECs) were obtained from the Institute of BeNa Biotechnology (Beijing, China). RMECs were cultured at 37°C and 5% CO_2_ in complete DMEM medium containing FBS (10% *v/v*), penicillin (1% *v/v*) and streptomycin (1% *v/v*).

### Transient Transfection

RMECs were transfected with TGR5 or PKCδ siRNA (RiboBio, China) at 80% confluence by using the riboFECT CP Transfection Kit (RiboBio, China) in accordance with the manufacturer’s protocol. Briefly, 120 μL Opti-MEM medium containing 12 μL riboFECT CP, 100 nM TGR5 siRNA, 100 nM PKCδ siRNA or 100 nM NC was premixed in a 6-well plate. Next, the cells were transduced for 48 h, and silence efficiency was evidenced by Western blot. The detailed siRNA information is provided in Supplementary Table S3.

### Animals and the DR Model

Male Sprague-Dawley rats of 6 weeks (220–250 g) were obtained from Changzhou Cavans Experimental Animal Co., Ltd. (China). All animal procedures were conducted in accordance with the National Institutes of Health Guide for the Care and Use of Laboratory Animals and approved by the Animal Care and Use Committee of Nanjing Medical University before implementation (approval number, SYXK(SU)2020-0010).

The rats were conducted by intraperitoneal injection of streptozotocin (STZ, 1.5%, 60 mg/kg). Diabetic rats were successfully established when blood glucose greater than 16.7 mM.

The diabetic retinopathy model was successfully established by streptozotocin (STZ) administration as previously described (Xin et al., 2012; Guo and Liu, 2017). In short, rats were fasted for 8 h with free access to water and then intraperitoneally injected with STZ (1.5%, 60 mg/kg) dissolved in 10 mM citrate buffer at pH 4.5. Meanwhile, the nondiabetic rats were treated with the same volume citrate buffer. 2 days after STZ treatment, the fasting plasma glucose (FPG) was sampled from the tail vein continuously for 3 days. The diabetic rat model was successfully established when blood glucose >16.7 mM for two consecutive measurements. Rats were housed for 18 weeks following induction to ensure the development of diabetic retinopathy. We observed retinal microvascular abnormalities in diabetic rats, including intraretinal leakage and hemorrhage, which were identified as pre-proliferative stage of diabetic retinopathy at week 18. Afterwards, rats were euthanized and the retinal tissues were obtained for further analyses.

### Intravitreal Injection

After 2 weeks, when the diabetic model was stably established, drug administration was initiated. The Mdivi-1 dose (30 ng/μL) and Rapamycin dose (5 ng/μL) were chosen according to the literature and preliminary studies (Zhu et al., 2020; Lee et al., 2021). Rats were anesthetised with the intraperitoneal injection of sodium pentobarbital (50 mg/kg). Ten µl Hamilton syringe (Hamilton, 7803–05) with a 33-gauge needle were used to perform intravitreal injections as described previously (Murakami et al., 2012). Mdivi-1 (30 ng/μL, 5 μL) or Rapamycin (5 ng/μL, 5 μL) was injected behind the superior temporal corneal limbus into the vitreous cavity by using a sterile 33-gauge needle every 4 weeks for a further 16 weeks. The eyes of rats in the nontreatment group were injected with an equivalent amount of solvent. After the injection, ofloxacin ointment was applied to the eyeball.

### TGR5 Knockdown in Rat by Adeno-Associated Virus Serotype 8

The AAV8 was constructed and packaged by the Vigene Biosciences and targeted the RNAi against TGR5 (AAV8-shTGR5) by controlling the albumin promoter. A 33-gauge needle was used to inject 5 μl AAV8-shTGR5 (6.25 × 10^12^ viral genomes/mL) gradually by intravitreal injection 2 weeks before streptozotocin (STZ) administration. The detailed shRNA information is provided in Supplementary Table S3 *.*Diabetic rats were randomly assigned into four groups: AAV8-vector, AAV8-shTGR5, AAV8-shTGR5 + Mdivi-1, and AAV8–shTGR5 + Rapamycin.

### CCK8 Assay

The CCK8 was used to examine cell viability in accordance with the manufacturer’s protocol (Beyotime, China). The cell suspension was inoculated in a 96-well plate (100 μL/well) at a minimum density of at least 1,000 cells per well. Then, cells were pretreated with drugs for 2 h and stimulated with or without HG (33 mM) for 5 days. Finally, 100 μL CCK-8 solution was added into each well for 1.5 h at 37°C, and absorbance values were measured at 450 nm by using a microplate reader.

### Analysis of Mitochondrial Morphology

Mitochondrial morphology was observed using MitoTracker staining (DOJINDO, Japan). Cells (1 × 10^4^) were incubated in 24-well plates for 5 days after drug treatment. The cells were stained with pre-warmed MitoTracker Red (200 nM, 30 min) at 37°C and 5% CO_2_ in the dark. After washing three times with PBS, live confocal imaging was performed to evaluate the mitochondrial morphology by confocal microscopy (63×, Leica, Germany) and the length of mitochondria was quantified with ImageJ software as described previously (Kang et al., 2020).

### Measurement of Intracellular Ca^2+^

Cells (1 ×10^4^) were incubated in 24-well plates for 3 days after drug treatment. Intracellular Ca^2+^ were measured with Fluo-4 AM (2 μM, Beyotime, China) for 1 h and then mitochondria were stained with the MitoTracker Red (200 nM; DOJINDO, Japan) for 30 min according to manufacturers’ instructions. The fluorescent intensity was obtained Colocalization of intracellular Ca^2+^ and mitochondria was detected by Laser scanning Confocal Microscopy (40×, Leica, Germany).

### Detection of Mitochondrial Membrane Potential

The JC-1 assay kit (Beyotime, China) is universally used to detect the mitochondrial membrane potential. JC-1 aggregated in the mitochondrial matrix at high membrane potential and could form red fluorescent aggregates. However, JC-1 cannot aggregate to form a polymer at low membrane potential and produce green fluorescent monomers. Briefly, cells (1 × 10^4^) were incubated into 24-well plates for 5 days after drug treatment. The cells were The JC-1 staining solution was added for 20 min, incubated with JC-1 dye at 37 °C for 20 min, washed twice with dilution buffer, and placed in the culture medium. and the fluorescence intensity was obtained by laser scanning confocal microscopy (40×, Leica, Germany) Lastly, images were obtained by laser scanning confocal microscopy (40×, Leica, Germany) at 490 nm excitation and 530 nm emission for green and at 525 nm excitation and 590 nm emission for red. Mitochondrial membrane potential is expressed as the ratio of red fluorescence (JC-1 aggregates) to green fluorescence (JC-1 monomers) using the ImageJ software.

### TUNEL Assay

RMECs (1 × 10^4^) were incubated in 24-well plates for 5 days after drug treatment. Cells or tissue sections were fixed with 4% paraformaldehyde for 30 min. After washing three times with PBS, triton-100 (0.2%) was added to samples for permeabilisation, and TUNEL detection solutions were reactions were incubated on ice for 5 min. After washing three times, TUNEL reaction mixture was prepared by mixing 50 µL Enzyme solution to 450 µL Label solution, 50 µL of TUNEL reaction mixture was added to sample and incubated for 1 h at 37°C in the dark. The samples were again washed three times with PBS and then nuclei were labelled with DAPI (1:1,000). TUNEL-fluorescent images were obtained by laser scanning confocal microscopy (40×, Leica, Germany). The TUNEL assay quantification was performed using the ImageJ software.

### Retinal Periodic Acid Schiff Staining

The amount of acellular capillaries was determined by retinal PAS staining according to the procedure previously described (Laver et al., 1993; Huang et al., 2015). The retina of rats was Briefly, the enucleated eyes were fixed with 4% paraformaldehyde for 24 h. Flat retinas were successfully isolated and fixed in 4% paraformaldehyde for 1.5 h and then digested with 3% trypsin for 2 h and samples were stained Nonvascular tissues were gently flushed away by a 1 ml syringe filled with PBS, and the visible vessels were mounted on glass slides under the microscope. After drying overnight, the samples were stained using the PAS/haematoxylin stain kit in accordance with the instructions (Solarbio, China). Degeneration of retinal capillaries can be was observed under light microscopy (Olympus, Tokyo, Japan).

### Permeability of Retinal BRB

As previously described (Leal et al., 2010), The integrity of the retinal BRB was accessed by Evans Blue dye. determined using the Evans Blue dye with minor modifications. The Evans Blue solution (3%) was injected into the tail vein of each rat under deep anaesthesia. After circulation for 2 h, rats were exsanguinated and perfused with ice-cold saline. After perfusion, the eyes were enucleated, the retinas were carefully dissected and mounted on glass slides, and observed under the BX-51 light microscope (Olympus, Tokyo, Japan).

### Immunohistochemical Staining

Firstly, samples were fixed with paraformaldehyde (4%, 30 min), permeabilised with Triton X100 (0.2%, 15 min) and blocked with QuickBlock™ Blocking Buffer (1 h). The primary antibodies p-Drp1 (1:100), HK2 (1:100), TOMM20 (1:50), PINK1 (1:100), isolectin B4 (IB4, 1:400), LC3B (1:500) and cleaved-caspase3 (1:400) and secondary antibody labelled by Alexa 488/555 were incubated into the samples. Nuclei were labelled with DAPI (1:1,000). Fluorescence images were observed using confocal microscopy (Leica, Germany).

### HE Staining

The assay followed a documented protocol (Koh et al., 2009). In brief, Firstly, paraffin sections of retinas were deparaffinised, dehydrated by gradient alcohol and subjected to HE staining. After dehydration, the sections were covered with neutral gum. The morphology of retinal tissue in rats were observed under the BX-51 light microscope (Olympus, Tokyo, Japan).

### Western Blot

RMECs were lysed with lysis buffer (Beyotime, China) containing protease, phosphatase inhibitors (Roche, Switzerland) and PMSF (Beyotime, China). The supernatant was obtained by centrifugation at 12,000 ×g for 15 min. Protein was quantified by a BCA kit (Beyotime, China). Protein samples were boiled in 1 × SDS-PAGE loading buffer (100°C, 5 min). The samples were electrophoresed and transferred onto PVDF, and the membranes were blocked with 5% milk. The primary antibodies TGR5 (1:1,000), Drp1 (1:1,000), p-Drp1 (1:1,000), PKCδ (1:2000), p-PKCδ (1:1,000), PINK1 (1:1,000), Parkin (1:1,000), HK2 (1:1,000), LC3B (1:1,000), P62 (1:5,000), BCL-2 (1:2000), Bax (1:2000), cleaved-caspase3 (1:1,000), Cox4 (1:5,000) and β-actin (1:5,000) were incubated overnight at 4°C. Then, horseradish peroxidase-linked antibodies were added for 2 h. Signal detection was visualised with a chemiluminescence kit (Thermo Fisher Scientific, United States).

### Quantification of Real-Time Polymerase Chain Reaction

The total RNA was extracted using Trizol, and reverse transcription was performed using the HiScript III RT SuperMix kit (Vazyme, China). The relative mRNA expression levels of OPA-1, Mfn-1, Mfn-2, Fis-1, and MFF were quantified by RT-PCR and normalised by GAPDH mRNA. Primer sequences are detailed in Supplementary Table S2.

### Statistical Analysis

Results were expressed as mean ± SD of three independent experiments. Datasets were compared by the Student t-test or single-factor ANOVA with Tukey’s post-hoc analysis. The GraphPad Prism version 8.00 (GraphPad Software, San Diego, CA) was used for data analysis. *p* < 0.05 was considered statistically significant.

## Results

### TGR5 Activation Alleviates HG-Induced Endothelial Cell Apoptosis

**FIGURE 1:**
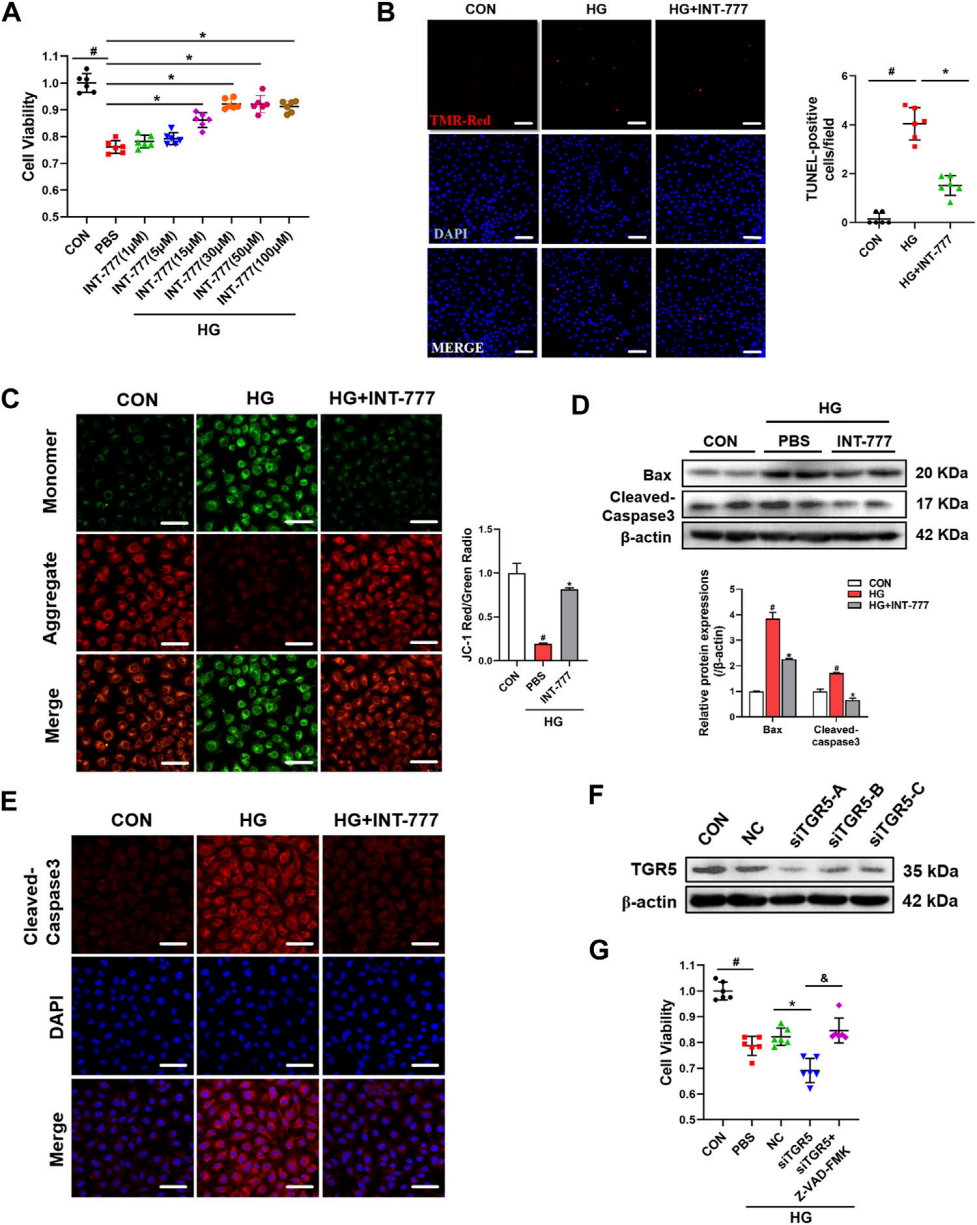
TGR5 activation alleviates high glucose (HG)-induced endothelial cell apoptosis. **(A–E)** RMECs were pretreated with INT-777 (30 μM) for 2 h and then exposed with or without HG (33 mM) for 5 days. **(A)** RMECs were pretreated with different dose of INT-777 (1, 5, 15, 30, 50, 100 μM) for 2 h and then exposed with or without HG (33 mM) for 5 days. Cell viability was measured using the CCK8 assay. **(B)** Cell apoptosis was measured by TUNEL staining kit and quantitative graphs show percentage of TUNEL positive cells. Scale bars = 50 μm. **(C)** Mitochondrial membrane potential was assessed by JC-1 staining. Quantification of the ratio of red fluorescence (JC-1 aggregates) to green fluorescence (JC-1 monomers). Sscale bars = 25 μm. **(D)** Apoptosis-related proteins were evaluated by Western blot. **(E)** Cleaved-Caspase3 was subjected to representative immunofluorescence analysis. Sscale bars = 25 μm. **(F)** RMECs were transduced with TGR5 siRNA for 48 h, and the transfection efficiency was evaluated by Western blot. **(G)** RMECs were transduced with TGR5 siRNA for 48 h and then incubated with Z-VAD (OMe)-FMK (10 μM) for 2 h followed by HG (33 mM) for 5 days. CCK8 assay was performed to determine cell viability. Results are presented as means ± SD. Data were analyzed by repeated measures (RM) ANOVA with Tukey’s HSD test. #*p* < 0.05 vs. control group, *p < 0.05 vs. HG group. &$ *p* < 0.05 vs. siTGR5 group.

Endothelial cell dysfunction is closely associated with the development of DR (Sabanayagam et al., 2019). Our previous studies have shown that INT-777, a TGR5 agonist, reduces retina damage by suppressing blood–retinal barrier (BRB) disruption in DR, but the mechanism of action remains unclear (Zhu et al., 2020). In the present study, we wondered whether the protective effect of TGR5 was associated with an improvement in endothelial function. Firstly, the CCK8 assay was used to evaluate cell viability (Figure 1A). The results revealed that the cell survival rate was significantly elevated in the INT-777-treated group compared with the HG-treated cells. dose-dependent increase of cell survival rates occurred with doses in the range 1–30 μM in HG + INT-777-treated groups. So we choose a dose of 30 μM for the follow-up experiment. Further analysis of the TUNEL staining showed a lower percentage of TUNEL-positive cells in the HG + INT-777-treated group than those in the HG-treated group (Figure 1B). The mitochondrial membrane potential is a critical indicator of mitochondrial function and regarded as one of the earliest events in the process of apoptosis. As shown in Figure 1C, INT-777 could reverse the reduction of mitochondrial membrane potential which resulted from HG.

To further confirm the inhibitory effect of INT-777 on endothelial cell apoptosis, Western blot was performed to detect the expression levels of apoptotic-related proteins, Bax and cleaved-caspase3. In the HG-treated group, the protein levels of Bax and cleaved-caspase3 were obviously high-expressed, compared with the control group. However, INT-777 could effectively protected part of cells from apoptosis caused by HG, suppressed the expression levels of apoptotic-related proteins. (Figure 1D). Similar results were observed with the immunofluorescence staining of cleaved-caspase3 (Figure 1E). Cells were transfected with TGR5-specific siRNA, and the successful knockdown efficiency was verified by Western blot assay (Figure 1F). The knocking down of TGR5 exacerbated HG-induced HRMEC injury, whereas the effect was partially blocked by Z-VAD-FMK, a well-known apoptotic inhibitor (Figure 1G). Overall, these results indicated that TGR5 was an important regulatory factor for the maintenance of endothelial cell function *in vitro*.

### TGR5 Regulates Mitochondrial Fission and Mitophagy Under HG Conditions

**FIGURE 2:**
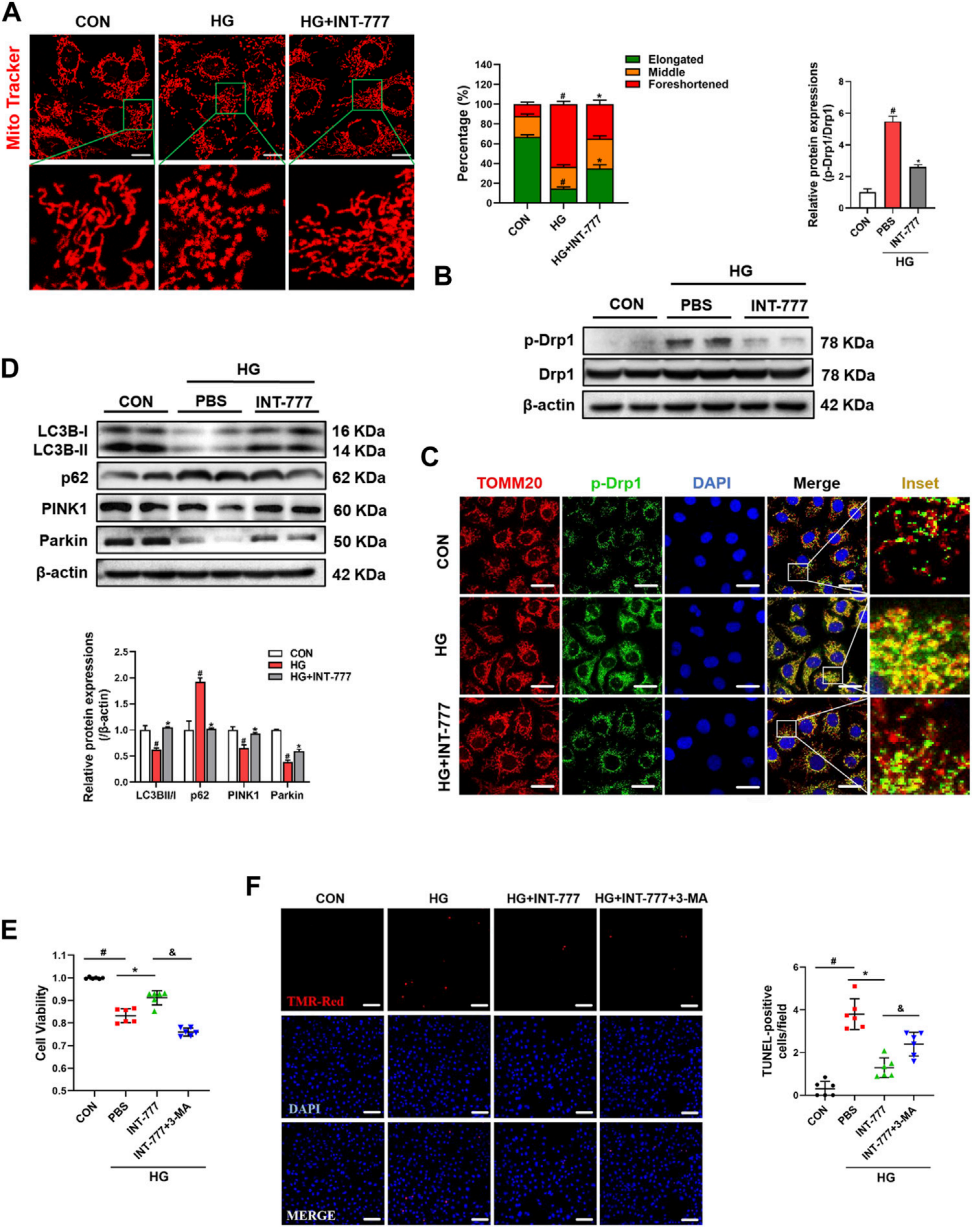
TGR5 regulates mitochondrial fission and mitophagy under HG conditions. RMECs were pretreated with INT-777 (30 μM) for 2 h and exposed with or without HG (33 mM) for **(A, E, F)** 5, or **(B, C, D)** 3 days. (F, G) RMECs were pretreated with INT-777 (30 μM) or INT-777 (30 μM) + 3-MA (5 mM) for 2 h and exposed with or without HG (33 mM) for 5 days. **(A)** Mitochondria were stained with MitoBright LT Red (200 nM, 30 min) and detected by confocal microscopy (63X_bar, 10 μm). Quantitation was performed in triplicate and scored into three categories: foreshortened, middle, and elongated mitochondria. **(B)** p-Drp1 and Drp1 protein levels were measured by Western blotting. **(C)** DAPI, p-Drp1 and TOMM20 were subjected to representative immunofluorescence analysis. Scale bar = 25 μm. **(D)** Western blot analysis showed the expression levels of LC3B, p62, Parkin, and PINK1. **(E)** Cell viability was measured using the CCK8 assay. **(F)** Cell apoptosis was measured by TUNEL staining and quantitative graphs show percentage of TUNEL positive cells. Scale bars = 50 μm. All data were presented as mean ± SD and analysed by RM ANOVA with Tukey’s HSD test. #*p* < 0.05 vs. control group, *p < 0.05 vs. HG group.

Dynamic mitochondrial processes, especially mitochondrial fission and mitophagy, play a key role in the pathogenesis of DR (Yamashita and Kanki, 2017; Ferrington et al., 2020). Firstly, we examined the morphological changes of mitochondria after HG treatment in RMECs. As shown in Figure 2A, cells had a fragmented mitochondrial network characterized by short rod-shaped mitochondria after HG stimulation, compared with control group. Conversely, INT-777 treatment restored the HG-induced mitochondrial morphological injury. To explore the TGR5 regulatory mechanism in mitochondrial dynamics, we detected the mRNA expression levels of mitochondrial fusion and fission by qPCR, including of OPA1, Mfn1, Mfn2, and MFF, Fis1 respectively. As shown in Supplementary Figure S1, HG-treated cells had significantly decreased transcriptional levels of Mfn1 and Mfn2 and increased Fis1 levels, compared with control group. Moreover, INT-777 did not reverse these alterations. Drp1 is a small GTPase that mediates mitochondrial fission. The phosphorylation of serine 616 in Drp1 increased its activity and further promoted mitochondrial fragmentation (Chen et al., 2019). Therefore, the p-Drp1 protein expression level was determined by Western blot. Our results demonstrated that HG increased p-Drp1 protein expression, which was reversed by INT-777 in RMECs (Figure 2B). Consistently, in immunofluorescence staining, we observed that p-Drp1 was recruited to the mitochondria and that INT-777 blocked the effect (Figure 2C). These results elaborated that TGR5 inhibited the Drp1-mediated mitochondrial fission.

Mitochondria were damaged by excessive mitochondrial fission, and damaged mitochondria were degraded by mitophagy. Next, we wondered whether TGR5 participated in the autophagy-dependent clearance of damaged mitochondria. As shown in Figure 2D, HG decreased the protein levels of LC3B Ⅱ/Ⅰ, PINK1, and Parkin and increased the protein level of p62, compared with control group. Conversely, INT-777 weakened these effects, suggesting that INT-777 could regulate mitophagy through the PINK1/Parkin pathway. However, 3-MA, an autophagic inhibitor, significantly prevented the anti-apoptosis effect of INT-777 (Figures 2E,F). These results revealed that TGR5 activated the PINK1/Parkin-mediated mitophagy.

### TGR5 Decreases Drp1 Phosphorylation by Inhibiting the Ca^2+^-PKCδ Pathway

**FIGURE 3:**
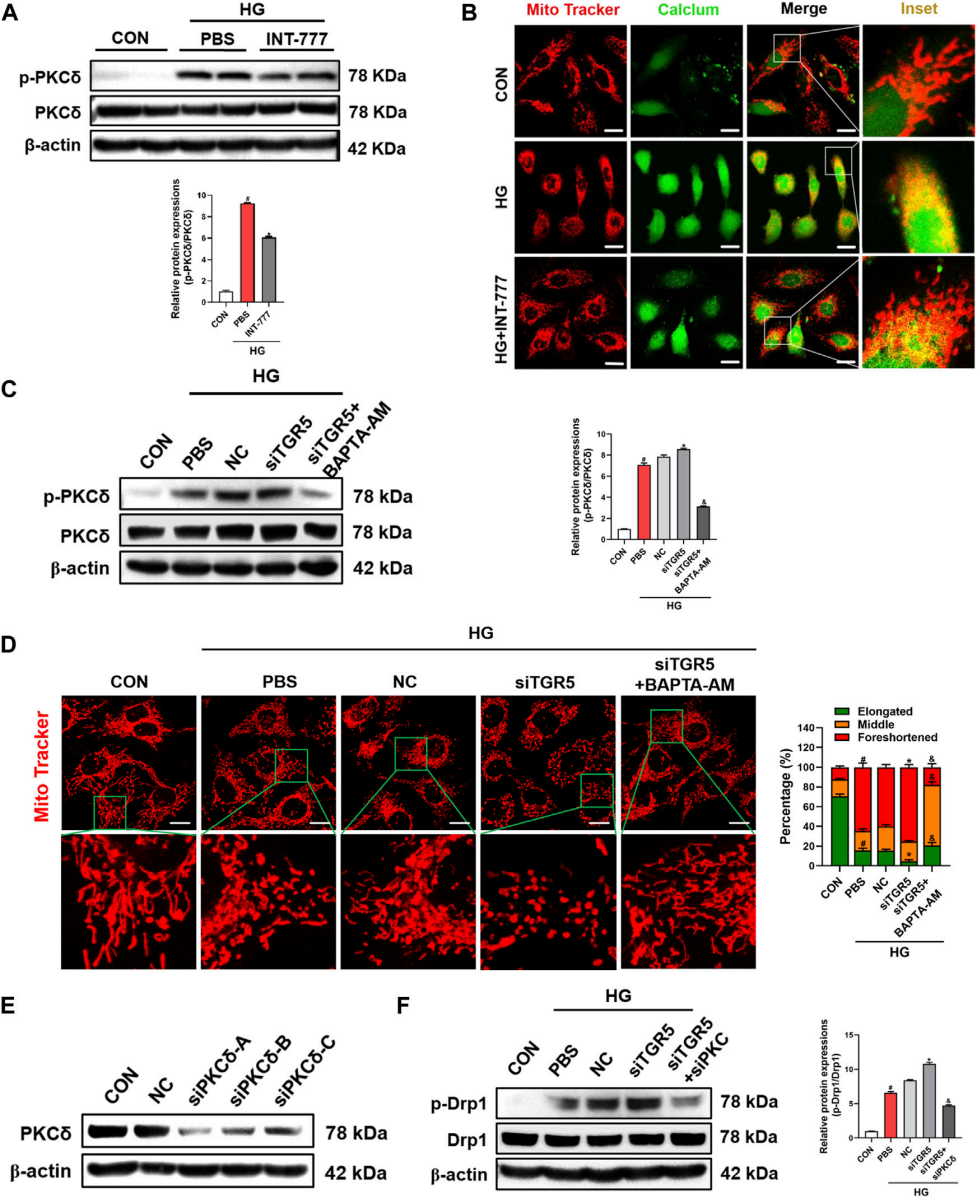
TGR5 decreases the Drp1 phosphorylation by inhibiting the Ca^2+^-PKCδ pathway. **(A, B)** RMECs were pretreated with INT-777 (30 μM) for 2 h and exposed with or without HG (33 mM) for 3 days. RMECs were transduced with TGR5 siRNA for 48 h and then incubated with BAPTA-AM (10 μM) for 2 h followed by HG (33 mM) for **(C)** three or **(D)** 5 days. **(E, F)** RMECs were transduced with TGR5 siRNA or PKCδ siRNA for 48 h and then incubated with HG (33 mM) for 3 days. **(A)** PKCδ phosphorylation level was detected by Western blotting. **(B)** Intracellular Ca^2+^ levels were detected by Fluo 4-AM (2 μM, 30 min) and mitochondria were stained with MitoBright LT Red (200 nM, 30 min). Colocalization of intracellular Ca^2+^ and mitochondria was detected by confocal microscopy. Scale bar = 25 μm. Intracellular Ca ^2+^ levels were detected by Fluo 4-AM (2 μ M, 30 min; scale bar = 25 μ m) . **(C)** Western blotting was conducted to determine the phosphorylation level of PKCδ. **(D)** Mitochondria were stained with MitoBright LT Red (200 nM, 30 min) and detected by confocal microscopy (63X_bar, 10 μm). Quantification of cells with different forms of mitochondrial morphology is shown in the bar chart. **(E)** PKCδ transfection efficiency was evaluated by Western blotting. **(F)** Drp1 phosphorylation levels were detected in different groups by Western blot. All data were presented as mean ± SD and analysed by RM ANOVA with Tukey’s HSD test. #*p* < 0.05 vs. control group, *p < 0.05 vs. HG or NC group, &$ *p* < 0.05 vs. siTGR5 group.

HG enhances intracellular Ca^2+^ concentration and PKC activation (Steinberg, 2008). Several researchers reported that the activated PKCδ can phosphorylate Drp1, further lead to its translocation to the mitochondria and promote mitochondrial fission (Zaja et al., 2014; Chen et al., 2019). Hence, we wondered whether the regulatory role of TGR5 in mitochondrial fission was associated with PKCδ. As shown in Figure 3A, HG increased the phosphorylation level of PKCδ. Interestingly, we found that the increase was blocked by TGR5 activation. Additionally, the influx of extracellular Ca^2+^ was drastically elevated by HG but blocked by INT-777, indicating that TGR5 might prevent mitochondrial fission by inhibiting the Ca^2+^-PKCδ/Drp1 signaling pathway (). colocalization of intracellular Ca^2+^ and mitochondria was drastically elevated by HG but blocked by INT-777, indicating that TGR5 might prevent mitochondrial fission by inhibiting the Ca^2+^-PKCδ/Drp1 signaling pathway (Figure 3B).

To verify whether TGR5 regulated mitochondrial fission through the Ca^2+^-PKCδ/Drp1 signaling pathway, we constructed the TGR5 siRNA for further in-depth studies. Our results showed that the siTGR5 treatment further increased the HG-induced p-PKCδ expression level in RMECs. Conversely, together treated with BAPTA-AM, a selective pharmacological inhibitor of Ca^2+^, significantly blocked the phosphorylation of PKCδ compared with siTGR5 group (Figure 3C). Consistent with these results, the mitochondrial morphology in siTGR5 group became more fragmented compared with that in NC group, and this phenomenon could be alleviated by BAPTA-AM (Figure 3D). Next, RMECs were transfected with PKCδ-specific siRNA (Figure 3E). As expected, the knocking down of PKCδ reversed siTGR5-induced overactivation of p-Drp1 (Figure 3F). The above experimental data indicated that TGR5 inhibited the mitochondrial fission by suppressing the Ca^2+^-PKCδ/Drp1 signaling pathway.

### Drp1 Inhibits Mitophagy by Facilitating the HK2 Separation From Mitochondria

**FIGURE 4:**
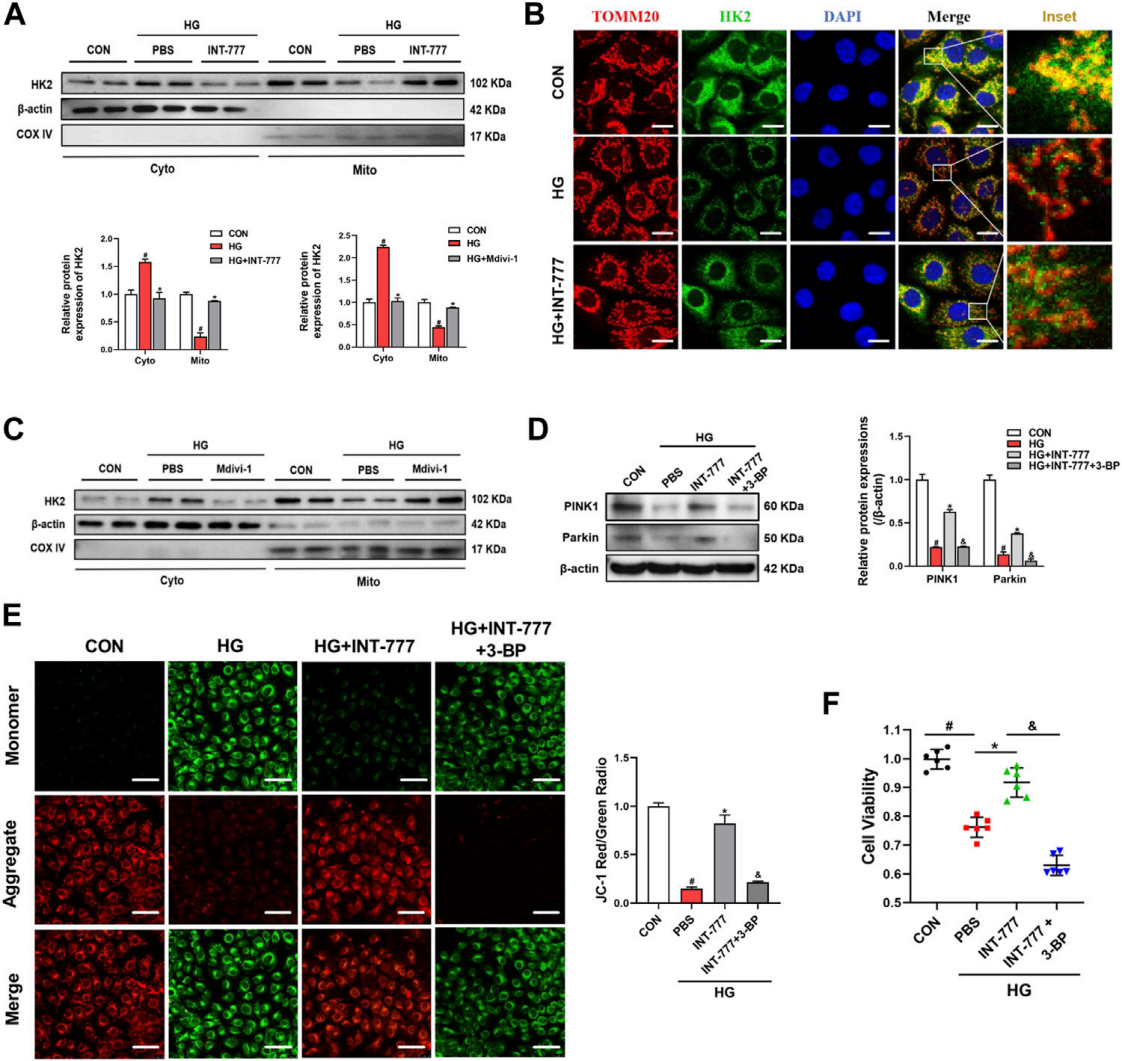
Drp1 inhibits mitophagy by facilitating HK2 separation from the mitochondria. RMECs were pretreated with **(A, B)** INT-777 (30 μM) or **(C)** Mdivi-1 (10 μM) for 2 h and then exposed with or without HG (33 mM) for 3 days. RMECs were pretreated with INT-777 (30 μM) or INT-777 (30 μM) + 3-BP (10 μM) for 2 h followed by HG (33 mM) for **(D, E)** three or **(F)** 5 days. **(A, C)** HK2 expression in mitochondrial and cytosolic fractions was detected by Western blot. All data were presented as mean ± SD and analysed by RM ANOVA with Tukey’s HSD test. #*p* < 0.05 vs. control group, *p < 0.05 vs. HG group. **(B)** DAPI, HK2 and TOMM20 were subjected to representative immunofluorescence analysis. S (scale bar = 25 μm. **(D)** Western blot analysis showed the protein expression levels of PINK1 and Parkin. **(E)** JC-1 dye was used to assess mitochondrial membrane potential in different groups. Quantification of the ratio of fluorescence intensity (red/green) is shown in the bar chart. S (scale bar = 25 μm). **(F)** Cell viability was measured using the CCK8 assay. All data were presented as mean ± SD and analysed by RM ANOVA with Tukey’s HSD test. #*p* < 0.05 vs. control group, *p < 0.05 vs. HG group, &$ *p* < 0.05 vs. INT-777 group.

Under the physiological states, mitochondrial fission can generate a small amount of damaged mitochondria, and damaged mitochondria are degraded by mitophagy (Schmukler et al., 2020). Our previous results indicated that TGR5 increased the PINK1/Parkin-mediated mitophagy and alleviated endothelial damage (Figures 2E–G). Next, we wondered the molecular mechanisms of the regulation of TGR5 on mitophagy. HK2 is a predominant HK isoform in insulin-sensitive tissues including the retinopathy. HK2 is a positive regulator of Parkin recruitment and tailors glycolysis (McCoy et al., 2014). Our results showed that HG caused HK2 translocation from the mitochondria to the cytosol and that INT-777 treatment could promote HK2 recruitment to mitochondria and further activate the PINK1/Parkin signaling pathway (Figures 4A,B). Thus, it can be concluded that TGR5 could reduce mitochondrial fission by suppressing the Ca^2+^-PKCδ/Drp1 signaling pathway and enhance mitophagy through the upregulation of the HK2-PINK1/Parkin signaling pathway. However, the relationship between mitochondrial fission and mitophagy still need to be explored. Next, we used Mdivi-1, a Drp1 inhibitor, to explore the underlying relationship between mitochondrial fission and mitophagy. Our results showed that Mdivi-1 promoted the translocation of HK2 from the cytosol to the mitochondria, suggesting the competitive binding of Drp1 and HK2 to the mitochondria (Figure 4C).

The HK2 inhibitor 3-bromopyruvate (3-BP) was used in this study to further confirm that TGR5 regulated PINK1/Parkin signaling pathway through HK2. We found that the upregulation of PINK1 and Parkin induced by INT-777 was reversed by 3-BP (Figure 4D). In parallel, compared with INT-777 group alone, 3-BP combined treatment significantly decreased the mitochondrial membrane potential (Figure 4E), and this finding was consistent with decreased cell survival (Figure 4F). These studies indicated that TGR5 regulated mitophagy through the HK2-PINK1/Parkin signaling pathway. Based on the above results, the exact mechanism of TGR5 regulating mitochondrial homeostasis was the Ca^2+^-PKCδ/Drp1-HK2-PINK1/Parkin signaling pathway.

### Inhibiting Mitochondrial Fission and Promoting Mitophagy Alleviate the AAV8-shTGR5-Induced Retinal Dysfunction *in Vivo*

**FIGURE 5:**
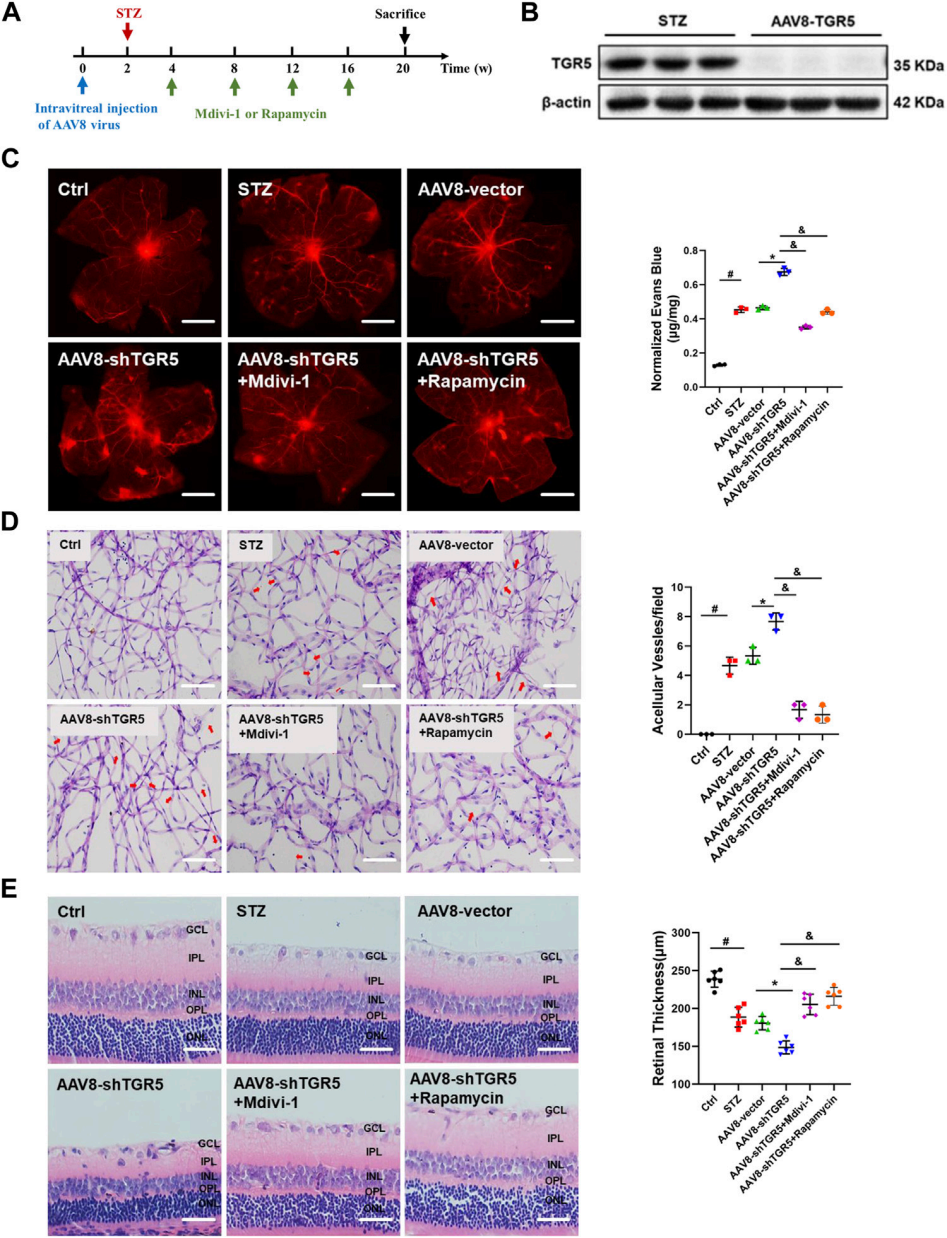
Mdivi-1 and Rapamycin alleviate the AAV8-shTGR5-induced retinal dysfunction *in vivo*. **(A)** Experimental workflow overview of *in vivo* experiments. **(B)** TGR5 shRNA lentivirus knockdown efficiency was assessed by Western blot. **(C)** Rats were infused with Evans Blue dye (3%) for 2 h. Red fluorescence dots indicate retinal vascular leakage in the flat-mounted retina by fluorescence microscopy. **(D)** Representative images of acellular capillaries were revealed by retinal trypsin digestion assay and quantified in random fields. (Sscale bar = 25 μm). **(E)** Representative images for haematoxylin–eosin (HE) staining in rat retinas. Retinal thickness was evaluated in sections. (Sscale bar = 25 μm). All data were presented as mean ± SD and analysed by RM ANOVA with Tukey’s HSD test. #*p* < 0.05 vs. control group, *p < 0.05 vs. AAV8-vector group. &$ *p* < 0.05 vs. AAV8-shTGR5 group.

The above results indicated that TGR5 inhibited mitochondrial fission and enhanced mitophagy by regulating the Ca^2+^-PKCδ/Drp1-HK2 signaling pathway To further confirm the role of TGR5 in DR, we performed an *in vivo* experiment by using AAV8-shTGR5 in DR rat to verify the hypothesis. As shown in Figure 5A, rats were pretreated by the intravitreal injection of AAV8-shTGR5 or AAV8-vector in the first week, and STZ (60 mg/kg) was intraperitoneally injected in the second week to establish an animal model of DR. Next, mitochondrial fission inhibitor Mdivi-1 (30 ng/μL, 5 μL) or the autophagy agonist Rapamycin (5 ng/μL, 5 μL) was injected by intravitreal injection every 3 weeks for 15 weeks in STZ-induced diabetic rats. The successful knockdown efficiency of TGR5 was verified by Western blot (Figure 5B). The intraretinal microvascular abnormalities (IRMA), including retinal vascular leakage and acellular capillaries, are classic hallmarks of DR. The retinal vascular leakage was observed in different retinal regions in DR rat. We found that DR-induced retinal vascular leakage was increased in the AAV8-shTGR5 group and significantly alleviated in groups pretreated with Mdivi-1 or Rapamycin (Figure 5C). The amount of acellular capillaries was determined by retinal trypsin digestion. Our results showed that capillary degeneration and pericyte loss in the AAV8-shTGR5 group were worse compared with those in the AAV8-vector group. Mdivi-1 or Rapamycin partially reversed this detrimental effect (Figure 5D). The retinal structural morphology was determined by HE staining. Our results showed that AAV8-shTGR5 aggravated STZ-induced retinal thickness loss, whereas the Mdivi-1 or Rapamycin groups significantly rescued the retinal thickness loss of the AAV8-shTGR5 group (Figure 5E).

**FIGURE 6:**
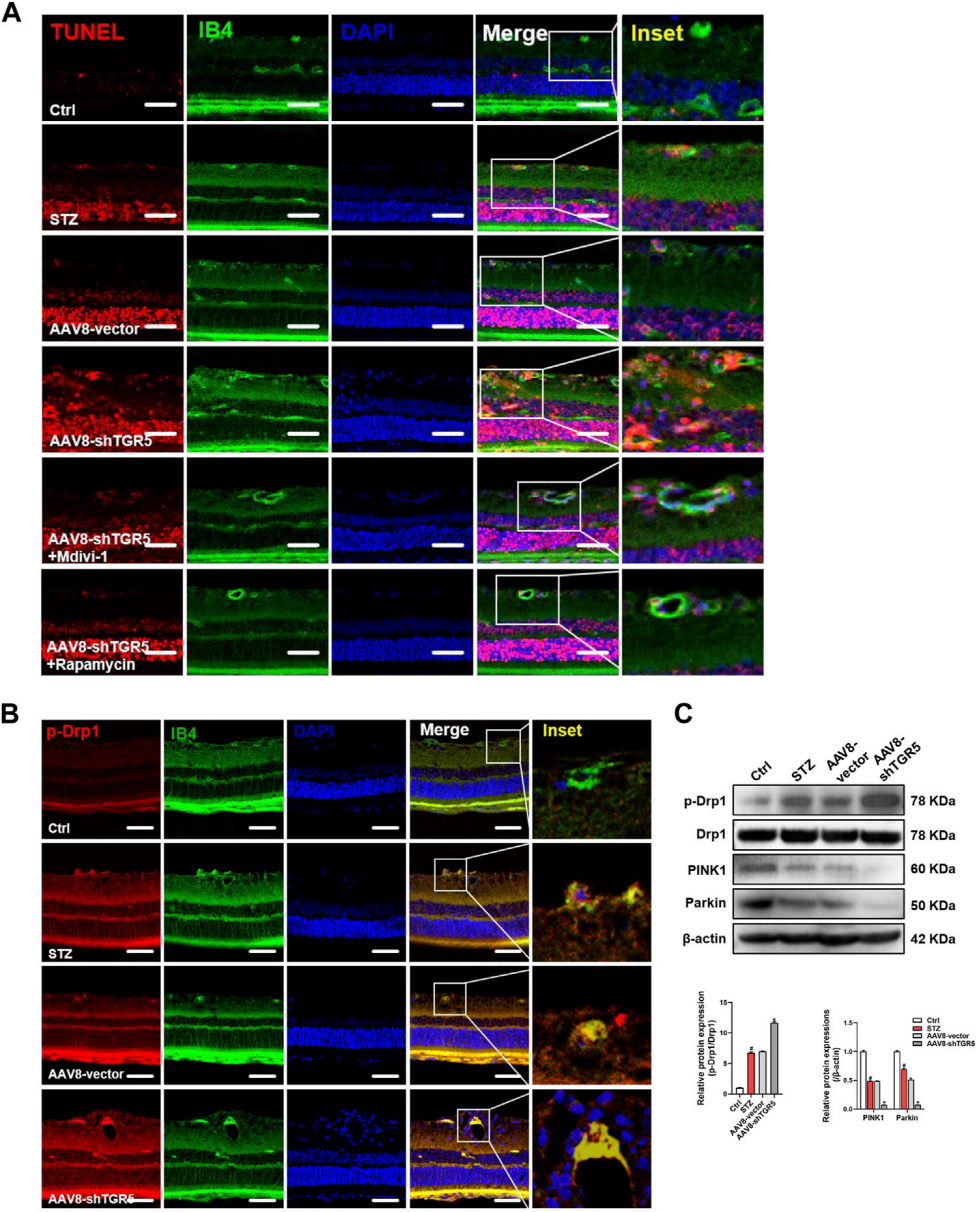
AAV8–shTGR5 aggravates STZ-induced mitochondrial dynamics disorder. **(A)** Apoptosis was assessed by TUNEL assay in retinal sections. Scale bar = 25 μm. **(B)** p-Drp1 (red), IB4 (green) and DAPI (blue) in retinal sections were subjected to immunofluorescence staining. Yellow fluorescence showed the colocalisation of green and red fluorescence. Scale bar = 25 μm. **(C)** Western blot analysis showed the protein expression levels of p-Drp1, Drp1, PINK1 and Parkin. All data were presented as mean ± SD and analysed by RM ANOVA with Tukey’s HSD test. #*p* < 0.05 vs. control group, *p < 0.05 vs. AAV8-vector group.

Endothelial cells undergoing apoptosis are associated with DR. The TUNEL assay is a classic methodology for apoptosis evaluation. AAV8-shTGR5 aggravated STZ-induced apoptosis, but Mdivi-1 or Rapamycin ameliorated the apoptosis worsened by AAV8-shTGR5 in DR rat (Figure 6A). Evidence showed that steady-state mitochondria occupied an important position in the development of DR, but its regulatory mechanism have not been verified . The relationship between mitochondrial dynamics and DR remains to be studied in the first step. The fluorescence colocalisation of p-Drp1 and IB4 (a vascular marker) was detected in the retinas of each group in DR rat (Figure 6B). In our study, results showed that diabetic rats displayed an augmented formation of the p-Drp1-IB4 complex compared with control rats, indicating that Drp1-induced mitochondrial fission might play a critical role in the progression of DR. Diabetic rats treated with AAV8-shTGR5 increased the colocalisation of p-Drp1 and IB4 compared with diabetic rats, and preliminary results showed that TGR5 inhibited mitochondrial fission by regulating Drp1. These effects were further supported by Western blot results (Figure 6C). Mitophagy also plays an important role in mitochondrial homeostasis. We detected mitophagy-related proteins (PINK1 and Parkin) *in vivo*. Our study showed that the AAV8-shTGR5 group decreased the protein levels of PINK1 and Parkin compared with AAV8-vector group (Figure 6C). These effects were further supported by IF results (Supplementary Figure S2A). Collectively, these results suggested that TGR5 ameliorated DR by affecting mitochondrial dynamics, including inhibited fission and enhanced mitophagy in RMECs.

## Discussion

**FIGURE 7:**
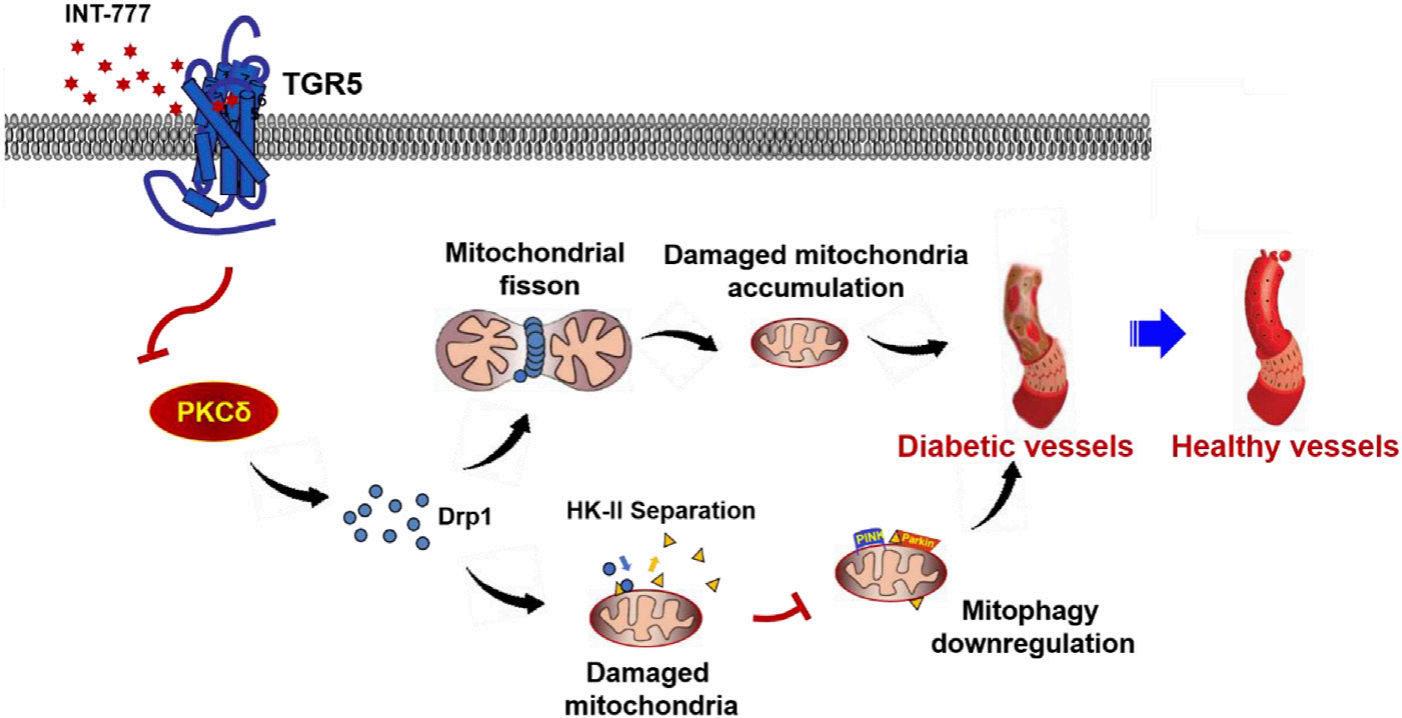
Possible mechanism for TGR5 alleviates DR by maintaining mitochondrial homeostasis. Figure 7 illustrated that TGR5 ameliorated DR by affecting mitochondrial dynamics, including inhibited mitochondrial fission and enhanced mitophagy in retinal endothelial cells. Specific regulatory mechanisms may be through regulating the PKCδ/Drp1-HK2-PINK1/Parkin signaling pathway. Our findings suggested that TGR5 may be a potential target for therapeutic intervention of DR.

RMECs are the primary target cells of HG-stimulated damage in DR. Previously, we showed that the activation of TGR5 may protect against DR development, but the underlying mechanism is unclear. In the present study, we confirmed that TGR5 ameliorated mitochondrial homeostasis by inhibiting mitochondrial fission and enhancing mitophagy throngh the PKCδ/Drp1-HK2-PINK1/Parkin signaling pathway, thereby improving endothelial dysfunction and alleviating DR progression (Figure 7).

Mitochondria are present in most eukaryotic cells and differ in size, number and morphology. As an indication of aerobic capacity, mitochondria account for up to 20–40% of the cell volume in cardiomyocytes, and the volume fraction is 7% in the endothelium cell (Yang et al., 2014). Notably, endothelial cells from different vascular beds have different mitochondrial contents. In blood-brain barrier endothelial cells, the mitochondrial content is as high as 8–11% (Carvalho et al., 2016). Endothelial cells rely on glycolysis for energy supply, leading to the perception that ATP originating from the mitochondria play no significant role in the endothelium. However, recent evidences showed that although the energy requirement between endotheliums is not as large as that between cardiomyocytes and smooth muscle cells, intracellular ATP likely plays important roles in mediating the normal physiological function of endothelial cells (Veronica Donoso et al., 2018). The mitochondrial oxidative phosphorylation serves an indispensable role for energy reserves. Moreover, the function of mitochondria is not limited to bioenergetics. Mitochondria play vital roles in regulating calcium homeostasis and reactive oxygen/nitrogen species production. The mitochondrial dysfunction can enhance oxidative stress sensitivity and lead to endothelial cell death. Thus, mitochondria are controlled by endothelium, and decreased mitochondrial function can also cause endothelial dysfunction.

Bile acids are primary metabolites which are converted from cholesterol in vertebrates. Bile acids are increasingly recognized as principal signaling factors of systemic endocrine functions and glucose metabolism homeostasis in addition to lipid homeostasis (Molinaro et al., 2018). Aberrant bile acid signaling involves the pathogenesis of multiple related diseases, including myocardial infarction, gallstone disease and DR (Chiang and Ferrell, 2019). Bile acids are synthesized in two ways, there are classical and alternative pathways, respectively. More than 90% of bile acids in the body are produced by the classical pathway. Cholesterol 7-α hydroxylase (CYP7A1), a major rate-limiting enzyme of the classic pathway, is expressed only in the liver (Ge et al., 2019). By contrast, bile acids synthesized by the alternative pathway occupies only 10% of the total amount of bile acids. Unlike the classic pathway, the alternative pathway is present in a wide range of extrahepatic tissues, including brain, skin and macrophages, which are regulated by the sterol 27 hydroxylase (CYP27A1) (Araya et al., 2003; Li and Chiang, 2014). Our research previously found that CYP27A1 in RMECs is significantly reduced after incubation under HG condition. Findings may suggest an unknown regulatory role for bile acid signaling in HG-mediated endothelial injury.

 We previously showed that TGR5 is expressed on RMECs and that TGR5 activation can regulate cell inflammatory responses and participate in the regulation of actin cytoskeletal remodelling by activating the cAMP-PKA signaling in RMECs, thereby preventing the process of DR. Consistent with our findings, the regulatory effect of TGR5 on PKA has been confirmed by literature (Hu et al., 2021; Jin et al., 2021). Interestingly, many documents showed that TGR5 is involved in the regulation of intracellular Ca^2+^ levels (Maczewsky et al., 2019; Bensalem et al., 2020; Halkias et al., 2021). Our present research found that TGR5 can stabilize endothelial cell mitochondrial dynamics and protect endothelial cells from HG damage. Besides, mitochondrial dynamics are partially associated with the activity of PKCδ (Serrat et al., 2013; Li et al., 2018), suggesting that TGR5 may regulate PKC activity by affecting intracellular Ca^2+^ levels and affecting mitochondrial homeostasis. In the present study, we found that INT-777 reduced PKCδ phosphorylation and blocked p-Drp1 expression. Our evidence suggested that endothelial dysfunction can be alleviated at least partially through TGR5/PKCδ/Drp1 signaling inhibition. 

 HK2 is the major HK subtype with high *in situ* rates of glucose metabolism, such as the brain, heart and skeletal muscle (Xu and Herschman, 2019). HK2 binds to the voltage-dependent anion channel and localizes to the outer mitochondrial membrane (Shuvo et al., 2016; Li et al., 2020). As an upstream regulator of mitophagy, HK2 known to positively regulate PINK1/Parkin aggregation Interestingly, we found that HG induced Drp-1 translocation from cytosol to mitochondria, thus squeezing HK-2 from mitochondria to cytosol and inhibiting mitophagy. Drp1 inhibitor promoted the translocation of HK2 from the cytosol to the mitochondria, and alleviated mitochondrial dysfunction. These results provided the first explanation of the molecular mechanism that the PKCδ/Drp1-HK2-PINK1/Parkin signaling pathway between mitochondrial fission and mitophagy. 

The above results indicated that TGR5 inhibited mitochondrial fission and enhances mitophagy *in vitro*. To further confirm the role of TGR5 in DR, we performed an *in vivo* experiment by using AAV8-shTGR5 in DR rat to verify our hypothesis. In our results, the TGR5 knockdown aggravated STZ-induced IRMA, including retinal vascular leakage, acellular capillaries and apoptosis in DR rats. Mdivi-1 or Rapamycin rescued the above phenomenon. *In vitro* and *in vivo* experiments documented that TGR5 regulated mitochondrial homeostasis by inhibiting mitochondrial fission, enhancing mitophagy and further attenuating DR progression.

Taken together, we showed that TGR5 inhibited mitochondrial fission and enhanced mitophagy in RMECs by regulating the PKCδ/Drp1-HK2-PINK1/Parkin signaling pathway, which revealed the molecular mechanisms underlying the protective effects of TGR5. However, our study also had the following limitations. Firstly, our results indicated that TGR5 decreases intracellular Ca^2+^ concentration by HG. Remarkably, intracellular calcium signaling is very delicate and complex, and conservation of intracellular Ca^2+^ is achieved by its redistribution between endoplasmic reticulum (ER) and mitochondria. The mechanism by TGR5 decreased mitochondrial calcium levels is less clear and perhaps ER calcium homeostasis play distinct functional roles in it, which need for further studies. Secondly, we have preliminarily found that TGR5 can affect mitochondrial autophagy, but the specific molecular mechanism was not deeply studied and a number of gaps are yet to be filled. Thirdly, the main conclusion of our study was only based on the role of TGR5 on endothelial cells, and further in-depth research is obviously required to elaborate DR pathogenesis precisely in future. Despite these limitations, we believe that our study helps to understand the imbalance of mitochondrial homeostasis is involved in DR, and TGR5 may serve as a potential therapeutic target for DR.

## Data Availability

The original contributions presented in the study are included in the article/Supplementary Material, further inquiries can be directed to the corresponding authors.

## Abbreviations

AAV8-shTGR5, Adeno-associated virus serotype 8-shTGR5; BRB, Blood-retinal barrier; DM, Diabetes mellitus; DR, Diabetic retinopathy; Drp1, Dynamin-related protein 1; Fis1, Mitochondrial fission protein 1; HG, High glucose; HK2, Hexokinase II; IB4, Isolectin B4; IRMA, Intraretinal retinal microvascular abnormalities; Mfn 1, Mitofusin 1; Mfn 2, Mitofusin 2; MFF, Mitochondrial fission factor; MQC, Mitochondrial Quality Control; OPA1, Optic atrophy1; PKCδ, Protein Kinase C delta type; RMECs, retinal vascular endothelial cells; ROS: Reactive oxygen species; STZ, Streptozotocin; TGR5, Bile Acid G-Protein-Coupled Membrane Receptor; VEGF, Vascular Endothelial Growth Factor
