# Supplementary material for: TGR5 Activation Ameliorates Mitochondrial Homeostasis via Regulating the PKCδ/Drp1-HK2 Signaling in Diabetic Retinopathy
Source: Front Cell Dev Biol. 2022 Jan 14;9:759421. doi: 10.3389/fcell.2021.759421 (PMC8795816; doi:10.3389/fcell.2021.759421)
Supplement: Supplementary file 1 [file DataSheet1.docx]

SUPPLEMENTARY MATERIAL

**Supplementary Table S1. Main materials and reagents**

| **Reagents** | **Source** | **Identifier** |
| --- | --- | --- |
| Glucose | Sigma-Aldrich | G8270 |
| 3-Bromopyruvic acid | MCE | [HY-19992](https://www.medchemexpress.cn/Ferrostatin-1.html) |
| Mdivi-1 | MCE | HY-15886 |
| INT-777 | MCE | HY-15677 |
| 3-Methyladenine | MCE | [HY-19312](https://www.medchemexpress.cn/Necrostatin-1.html) |
| Rapamycin | MCE | [HY-10219](https://www.medchemexpress.cn/Ruxolitinib.html) |
| BAPTA-AM | MCE | [HY-](https://www.medchemexpress.cn/fludarabine.html)100545 |
| Z-VAD(OMe)-FMK | MCE | HY-16658 |
| MitoBright LT Red | DOJINDO | MT11 |
| Fluo-4 AM | Beyotime | S1060 |
| Cell counting kit-8 | Beyotime | C0039 |
| JC1 assay kit | Beyotime | C2006 |
| Cell mitochondria isolation kit | Beyotime | C3601 |
| TUNEL assay kit | Roche | 12156792910 |
| HE staining kit | BOSTER | AR1180 |
| [Fetal Bovine Serum](https://www.thermofisher.com/cn/zh/home/life-science/cell-culture/mammalian-cell-culture/fbs.html?SID=fr-fbs-main) | Gibco | 10099-141 |
| Trypsin-EDTA (0.25%) | Gibco | 25200072 |
| Dulbecco's Modified Eagle Medium | Gibco | C11995500BT |
| riboFECT CP Transfection Kit | Ribo | C10511-1 |
| Mouse monoclonal anti-Parkin | CST | #4211 |
| Rabbit monoclonal anti-PINK1 | CST | #6949 |
| Rabbit monoclonal anti-Phospho-DRP1 | CST | #4494 |
| Rabbit monoclonal anti-Cleaved Caspase-3 | CST | #9661 |
| Rabbit monoclonal anti-HKII | Abcam | ab209847 |
| Rabbit monoclonal anti-PKCδ | Abcam | ab182126 |
| Rabbit monoclonal anti-Phospho-PKCδ | Abcam | ab76181 |
| Rabbit monoclonal anti-LC3B | Abcam | ab192890 |
| Rabbit monoclonal anti-P62 | Abcam | ab109012 |
| Rabbit monoclonal anti-DRP1 | Abcam | ab184247 |
| Rabbit polyclonal anti-Bcl2 | Proteintech | 12789-1-AP |
| Rabbit polyclonal anti-Bax | Proteintech | 50599-2-Ig |
| Rabbit polyclonal anti-COX4 | Proteintech | 11242-1-AP |
| Mouse monoclonal anti-β-actin | Proteintech | 66009-1-Ig |
| Mouse monoclonal anti-Tom20 | Santa | sc-17764 |
| HRP-labeled Goat Anti-Mouse IgG(H+L) | Beyotime | A0216 |
| HRP-labeled Goat Anti-Rabbit IgG(H+L) | Beyotime | A0208 |
| Goat anti-Rabbit IgG (H+L), Alexa Fluor 594 | Thermo Fisher | A11012 |
| DAPI | Thermo Fisher | R37606 |
| ECL kit | Thermo Fisher | 34580 |
| HiScript III RT SuperMix kit | Vazyme | R323-01 |
| Fast SYBR Green Master Mix | Roche | 04913914001-1 |

**Supplementary Table S2. Primers for Quantitative Real-Time PCR**

| Primer | Sequence |
| --- | --- |
| Human OPA-1 Forward | ACCGGACCTTAGTGAATATAAATGG |
| Human OPA-1 Reverse | TTCTTCCGGAGAACCAAAATCG |
| Human Fis-1 Forward | AGGCCTTAAAGTACGTCCGC |
| Human Fis-1 Reverse | ACAGCAAGTCCGATGAGTCC |
| Human Mfn-1 Forward | GCTGGCTAAGAAGGCGATTACT |
| Human Mfn-1 Reverse | CTCCGAGATAGCACCTCACC |
| Human Mfn-2 Forward | GTCTGACCTGGACCACCAAG |
| Human Mfn-2 Reverse | TGCAGTTGGAGCCAGTGTAG |
| Human MFF Forward | GCGAATGAGGGTCCCAGAAA |
| Human MFF Reverse | AGGTGGTGTTTTCAGTGCCA |

**Supplementary Table S3. The sequences of the siRNA (Human) and shRNA (Rat)**

| **Product number** | **Product name** | **Serial number** |
| --- | --- | --- |
| stB0004577A | si-PKCδ-001 | GCTTCAAGGTTCACAACTA |
| stB0004577B | si-PKCδ-002 | GCAAGTGCAACATCAACAA |
| stB0004577C | si-PKCδ-003 | GAAGCAGGGATTAAAGTGT |
| siG1466101050 | si-TGR5-001 | GTCTGGCATTGCCCACATT |
| siG1466101059 | si-TGR5-002 | CCTGTACCTCGAAGTCTAT |
| siG141217140319 | si-TGR5-003 | TCTACTTGGCTCCCAACTT |
| siB161011044323  pHS-ASR-0062 | NC-siRNA  TGR5-shRNA (rat) | GGCTCTAGAAAAGCCTATGC  GGGCCTGTAACTCTGTTATCT |
| pHS-ASR-LW416 | NC shRNA(rat) | TTCTCCGAACGTGTCACGTTT |


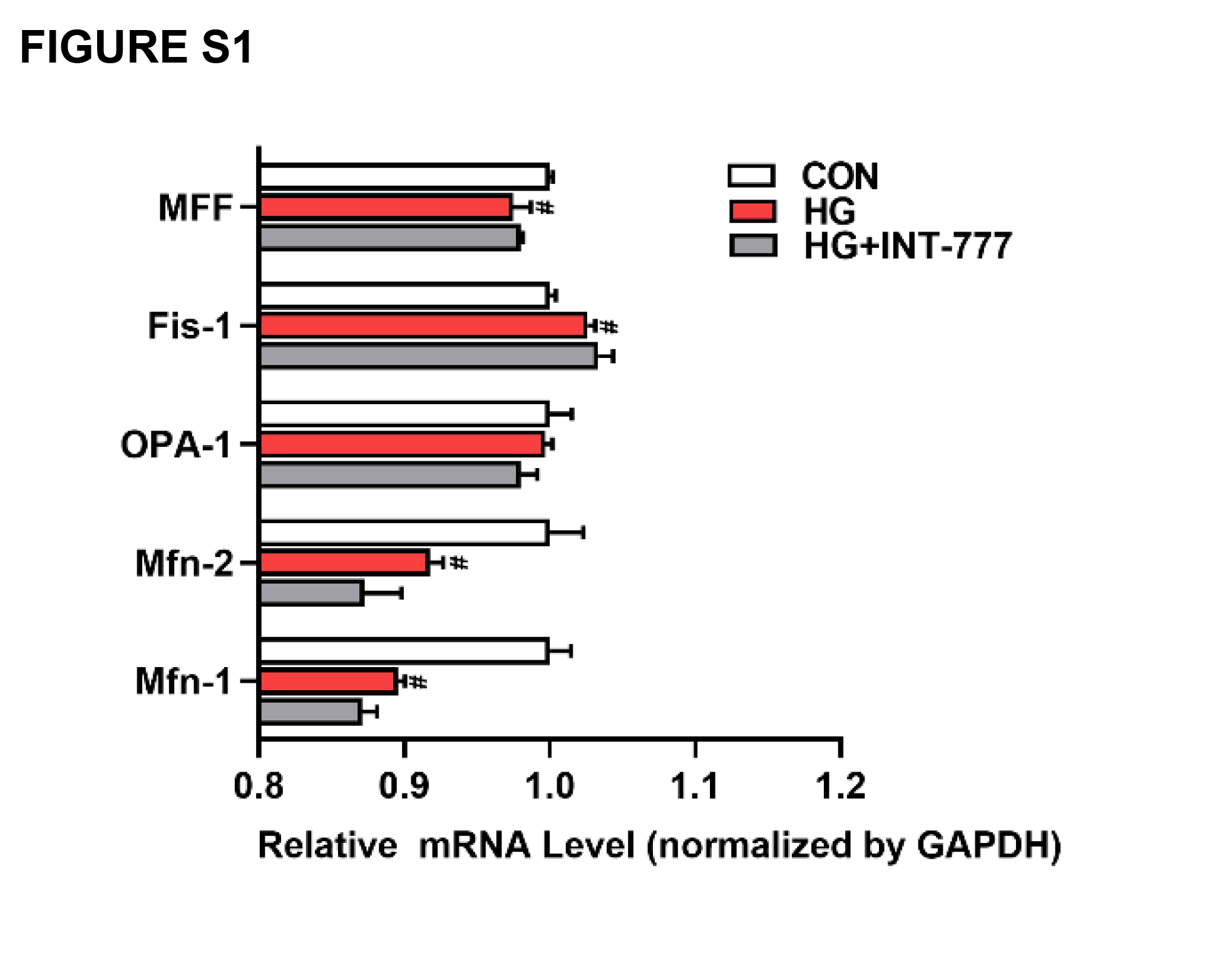


Supplementary Figure S1 OPA-1, Mfn-1, Mfn-2, MFF and Fis-1 mRNA expression levels were detected by RT-PCR.


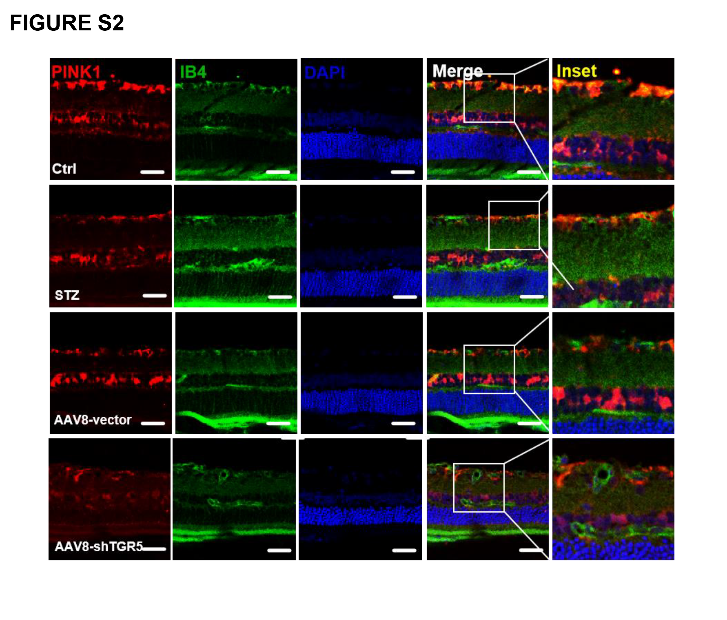


Supplementary Figure S2 The immunofluorescent staining of PINK1(Red), IB4 (Green) and DAPI (blue) in the retinal sections. scale bar=50 μm.
